# Maximum likelihood pandemic-scale phylogenetics

**DOI:** 10.1038/s41588-023-01368-0

**Published:** 2023-04-10

**Authors:** Nicola De Maio, Prabhav Kalaghatgi, Yatish Turakhia, Russell Corbett-Detig, Bui Quang Minh, Nick Goldman

**Affiliations:** 1 European Molecular Biology Laboratory, European Bioinformatics Institute (EMBL-EBI), Hinxton, UK; 2 Max Planck Institute for Molecular Genetics, Berlin, Germany; 3 Department of Electrical and Computer Engineering, University of California San Diego, San Diego, CA USA; 4 Department of Biomolecular Engineering, University of California Santa Cruz, Santa Cruz, CA USA; 5 Genomics Institute, University of California Santa Cruz, Santa Cruz, CA USA; 6 School of Computing, College of Engineering, Computing and Cybernetics, Australian National University, Canberra, Australian Capital Territory Australia

**Keywords:** Genome informatics, Microbial genetics, Genomics, Molecular biology, Software

## Abstract

Phylogenetics has a crucial role in genomic epidemiology. Enabled by unparalleled volumes of genome sequence data generated to study and help contain the COVID-19 pandemic, phylogenetic analyses of SARS-CoV-2 genomes have shed light on the virus’s origins, spread, and the emergence and reproductive success of new variants. However, most phylogenetic approaches, including maximum likelihood and Bayesian methods, cannot scale to the size of the datasets from the current pandemic. We present ‘MAximum Parsimonious Likelihood Estimation’ (MAPLE), an approach for likelihood-based phylogenetic analysis of epidemiological genomic datasets at unprecedented scales. MAPLE infers SARS-CoV-2 phylogenies more accurately than existing maximum likelihood approaches while running up to thousands of times faster, and requiring at least 100 times less memory on large datasets. This extends the reach of genomic epidemiology, allowing the continued use of accurate phylogenetic, phylogeographic and phylodynamic analyses on datasets of millions of genomes.

## Abstract (web summary)

‘MAximum Parsimonious Likelihood Estimation’ (MAPLE) is a maximum likelihood-based approach for inference of phylogenetic trees from very large datasets of similar sequences incorporating a sparse alignment representation and parsimony-based approximations, offering higher accuracy and reduced computational requirements.

## Main

As viruses and bacteria spread within and between hosts, they accumulate genetic mutations. By analyzing the genetic data of sampled pathogens, we can understand their evolutionary and transmission history. For this reason, genomic data have a crucial role in epidemiology, as exemplified during the COVID-19 pandemic, and are used to track and reconstruct the spread of disease within communities and within and between countries^1–6^, understand the dynamics of transmission^5,7–9^, estimate the efficacy of containment measures^10–13^ and predict future epidemiological dynamics^4,14^, and for the tracking of pathogen evolution as showcased by the identification of new SARS-CoV-2 mutations and variants of concern^15–19^.

Investigations of genomic epidemiological data are predominantly based on phylogenetic methods, but analyses of SARS-CoV-2 genome sequence data with existing phylogenetic approaches are becoming more difficult due to the excessive computational resources required by current global datasets consisting of millions of genomes^20^. Large and up-to-date global phylogenies^21^ are expected to be more accurate than smaller ones^22^ and allow detailed analyses such as for transmission tracking^6^ and lineage assignment^19^. However, estimating such large phylogenies accurately with established phylogenetic software like RAxML^23^ or IQ-TREE^24^ would require years for each tree update (if possible at all due to memory demand). For this reason, tools for tracking viral genome evolution and spread (for example NextStrain^25^) and many other genomic analyses often downsample global SARS-CoV-2 datasets to a few thousand genomes, leading to loss of power and resolution^22,26^.

### Results

#### Pandemic-scale likelihood-based phylogenetics

To address these issues, we have devised a set of algorithms, techniques and formats tailored for large-scale genomic epidemiology. Our approach, ‘MAximum Parsimonious Likelihood Estimation’ (MAPLE), performs maximum likelihood phylogenetic inference^23,24,27^ and uses explicit probabilistic models of sequence evolution; we combine these best-in-class features with some aspects of maximum parsimony methods^28^ that allow it to greatly reduce computer memory and time demand.

#### Concise genome data representation

Genomic data typically need to be aligned before performing phylogenetic inference; resulting alignments usually employ Fasta or similar formats^29^, which list the whole DNA sequence of each considered sample. In the context of genomic epidemiology, this is very highly redundant because genomes within an epidemic are usually extremely similar to each other. The VCF format can sometimes reduce alignment file size; however, with large datasets, as the number of variable sites approaches genome size, the VCF format can also become memory-demanding. While it is possible to reduce the size of datasets using standard compression techniques^30^, sequences still need to be uncompressed before analysis.

**Fig. 1:**
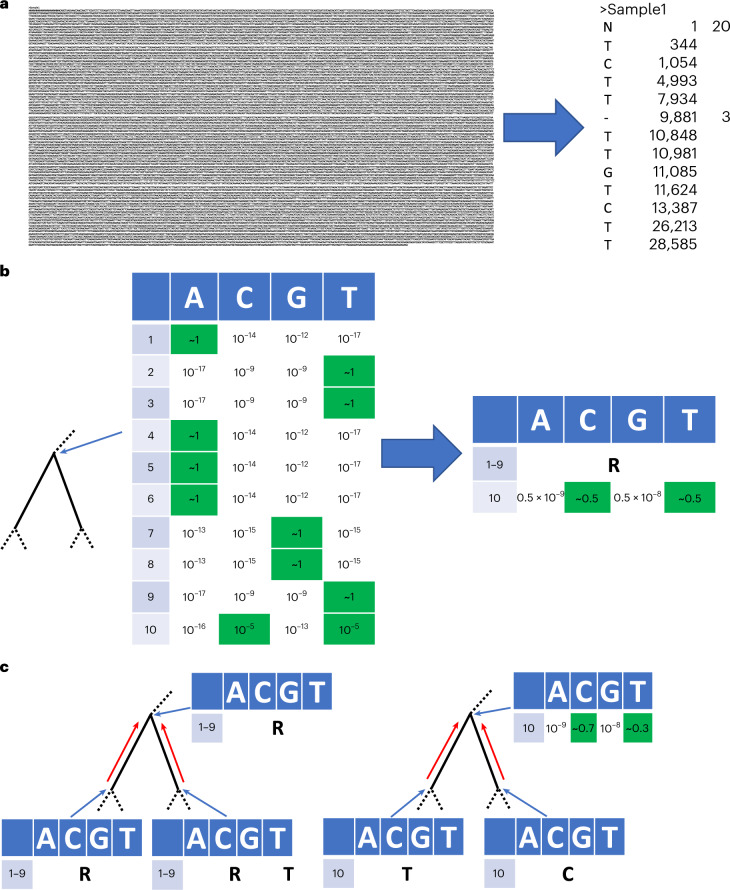
Graphical summary of sequence and likelihood representation and processing. **a**, Left—Fasta representation of an individual SARS-CoV-2 genome consists of sample name followed by the entire ≈ 30 kbp genome sequence. Right—MAPLE format records only the differences between the genome under consideration and a reference; columns represent the variant character observed, the position along the genome and (when necessary) the number of consecutive positions for which the character is observed. **b**, Left—an example likelihood vector at an internal node of a phylogenetic tree (shown by the narrow blue arrow; only a small portion of the tree is shown); for simplicity, we show only ten genome positions. At each position (rows 1–10), each column contains the likelihood for a specific nucleotide. For rows 1–9, the likelihood is concentrated at only one nucleotide (highlighted in green), while for position 10, we show an example with more uncertainty. Right—MAPLE representation of these node likelihoods. Assuming that the reference sequence at the first nine positions matches the most likely nucleotides in the vector (ATTAAAGGT), then for positions 1–9, the likelihood of nonreference nucleotides is negligible and we represent the likelihoods with a single symbol (R). At position 10, due to non-negligible uncertainty, we explicitly calculate and store the four relative likelihoods. **c**, Examples of likelihood calculation steps in MAPLE. Red arrows represent the flow of information from the tips to the root of the tree. Left—if two child nodes are in reference state R for a region of the genome (here, positions 1–9), then MAPLE assumes that their parent is also in state R. Right—if at a genome position (here, position 10), two child nodes have likelihoods concentrated at different nucleotides, then for their parent, we explicitly calculate the relative likelihoods of all four nucleotides.

**Fig. 2:**
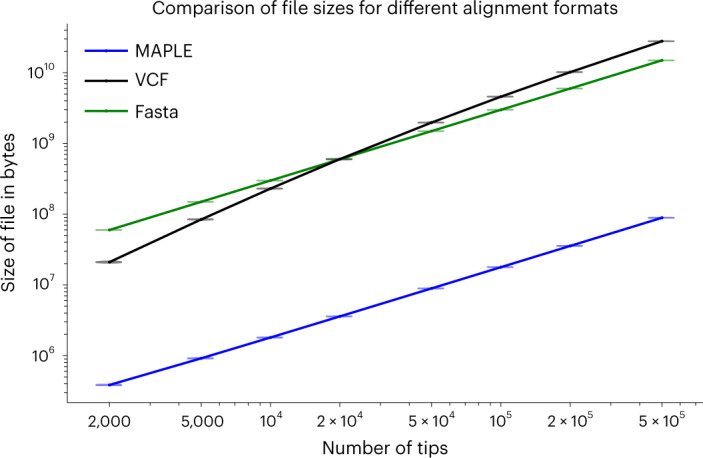
Comparison of file sizes of SARS-CoV-2 genome alignments using different alignment formats. On the *y* axis on a logarithmic scale, we show the sizes of alignment files for each format considered, expressed in bytes. On the *x* axis is the number of sequences in the dataset considered on a logarithmic scale. Here we consider random subsamples of our real SARS-COV-2 alignment data. Violin plots (often variation within one plot is not visible, collapsing the violin plots into horizontal lines) summarize values for 20 replicates, and dots represent their mean.

Instead, we represent each genome in our MAPLE alignment format in terms of differences with respect to a reference genome (Fig. 1a; Methods). This way, we reduce file size approximately 100-fold compared to Fasta files (Fig. 2); for example, we reduced the size of the 31-03-2021 GISAID global SARS-COV-2 alignment of 915,508 genomes from 27.84 GB to 224.6 MB (a 124× reduction).

#### Concise phylogenetic likelihoods

Likelihood-based phylogenetic methods typically keep track of the probability of every possible nucleotide at each position of the genome and each node of the phylogenetic tree^31,32^. With pandemic-scale genomic data, this process requires excessive computational time and memory resources^20^. However, in genomic epidemiology, due to the similarity of the genomes considered, these probabilities are typically highly concentrated at only one of the four nucleotides for most genome positions and tree nodes. We exploit this feature by approximating these probabilities and representing them concisely (Fig. 1b; Methods). As an example, when estimating a phylogeny from a random 10,000-sample subset of the GISAID dataset above, with a reference genome of 29,891 bp, on average we only record the phylogenetic likelihoods of 2.7 genome positions per tree node (≈10,000 times less than usual). This allows us to considerably reduce the memory demand of likelihood-based phylogenetic inference in genomic epidemiology.

Additionally, we develop a faster and approximate alternative to the Felsenstein pruning algorithm^32^ used to calculate phylogenetic likelihoods; this algorithm has been at the core of most of the likelihood-based phylogenetics in the past 40 years, and so is fundamental to some of the most cited and used scientific software, but is not tailored for the features of pandemic-scale genomic data. Our alternative (Fig. 1c; Methods) takes advantage of the strong similarities between the considered genomes and of concise likelihood and data representation to reduce the computational time demand of approximate likelihood-based phylogenetics in genomic epidemiology.

#### Fast tree exploration

**Fig. 3:**
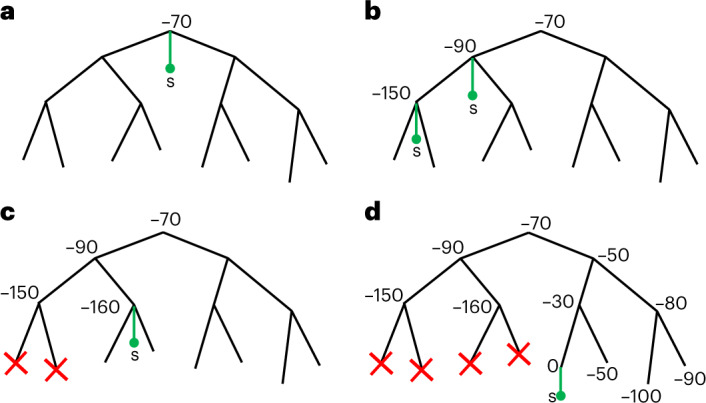
Graphical summary of phylogenetic placement in MAPLE. **a**, To search for the best placement of a new sample *s* (here represented by a green dot and branch) on the current tree, we first assess placement at the root, which in this case results in a relative log-likelihood score of −70. **b**, We iteratively visit descendant nodes by preorder traversal and assess placement for each visited node (in practice, we also attempt placement onto branches). **c**, When the log-likelihood score decreases two times consecutively and falls below a certain threshold relative to the best placement found so far, we do not visit further nodes downstream (red crosses). **d**, The placement with the highest score at the end of this process (in this case with cost 0) is taken as optimal for the addition of *s* to the tree.

To quickly but accurately find likely phylogenetic trees, we develop heuristic strategies for exploring tree space. Our first strategy is an adaptation of stepwise addition^33^, in which samples are added to the phylogenetic tree one at a time. We use this strategy to find an initial tree (which is then refined with the second strategy), but it is similarly useful in extending an existing tree, for example, as new genomes become available with time. Our adaptation involves a fast and approximate search among the nodes of the tree for the most likely tree position in which to add the new sample (Fig. 3; Methods).

Our second strategy consists of a modification of subtree pruning and regrafting^33^, which is used to perturb (and thereby improve) an existing tree. Our modification consists again in quickly exploring a broad range of possible tree changes.

#### Computational demand and accuracy of MAPLE

**Fig. 4:**
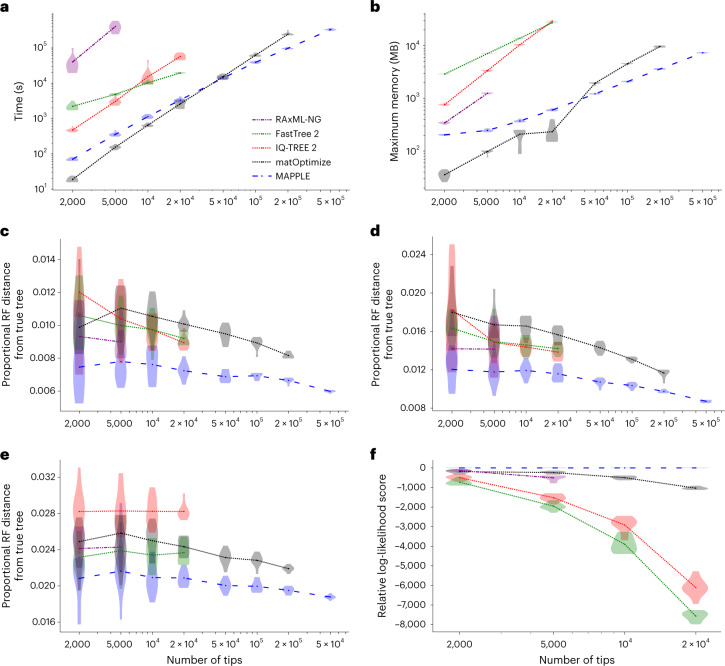
MAPLE consistently delivers higher accuracy phylogenetic inference from SARS-CoV-2 genomes at lower computational demand. **a**, Time required to run each method considered on real SARS-CoV-2 datasets. Each phylogenetic inference method considered is represented by a different color and line style (see legend). Values on the *x* axis show the number of samples included in each replicate. We ran each method up to the maximum dataset size that could be analyzed due to time (1 week) and memory (40 GB) limitations. Each violin plot summarizes values for ten replicates, and dots represent mean values. **b**, Maximum RAM demand required to run each method considered on real SARS-CoV-2 datasets. **c**–**e**, Proportional Robinson–Foulds (RF) distances between estimated trees and true trees in simulations. Higher values correspond to more errors in phylogenetic estimation. **c**, ‘Basic’ simulation scenario; **d**, ‘rate variation’ simulation scenario; **e**, ‘sequence ambiguity’ simulation scenario. **f**, Log-likelihoods (computed with IQ-TREE 2) of phylogenies inferred by different methods on real SARS-CoV-2 data, relative to the highest log-likelihood score obtained by any method for the same replicate. Higher values on the *y* axis represent more likely estimates. We consider only datasets of up to 20,000 samples due to the computational demand of likelihood evaluation.

**Extended Data Fig. 1:**
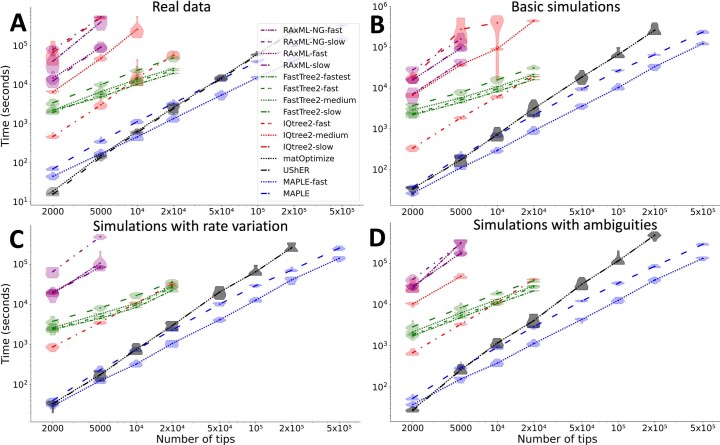
Comparison of running times of all considered methods and options for phylogenetic inference from SARS-CoV-2 genomes. Comparison of running times of all considered methods and options for phylogenetic inference from SARS-CoV-2 genomes. On the Y axis on a logarithmic scale, we show the number of seconds it takes to run each method. On the X axis is the number of sequences in the dataset considered on a logarithmic scale. Different line styles and colors represent different options for each method, as denoted in the legend and Supplementary Methods Section S1.11. We ran each method and set of options up to the maximum dataset size that was achievable due to time and memory limitations. Violin plots summarize values for 10 replicates, and dots represent their mean. **A** Results for subsamples from the real SARS-CoV-2 dataset. **B** Simulated datasets with no rate variation or ambiguity. **C** Results on simulated data with rate variation but no ambiguities. **D** Simulated data with sequence ambiguities but no rate variation.

**Extended Data Fig. 2:**
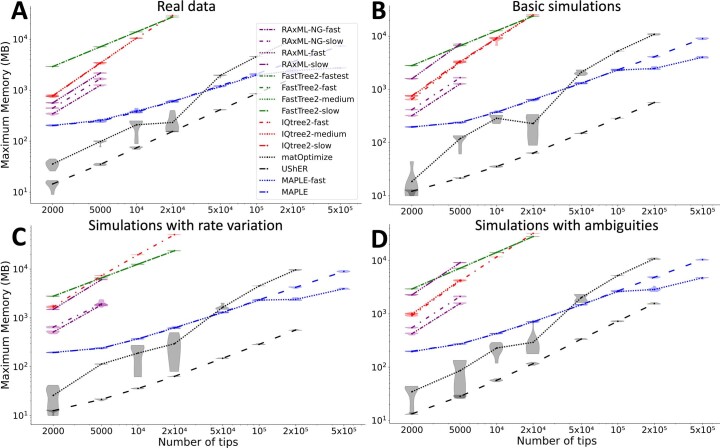
Comparison of maximum memory demand of all considered methods and options for phylogenetic inference from SARS-CoV-2 genomes. On the Y axis on a logarithmic scale, we show the maximum RAM memory demand in MB required to run each method. **A** Results for subsamples from the real SARS-CoV-2 dataset. **B** Simulated datasets with no rate variation or ambiguity. **C** Results on simulated data with rate variation but no ambiguities. **D** Simulated data with sequence ambiguities but no rate variation. Other details are the same as in Extended Data Figure S1.

**Extended Data Fig. 3:**
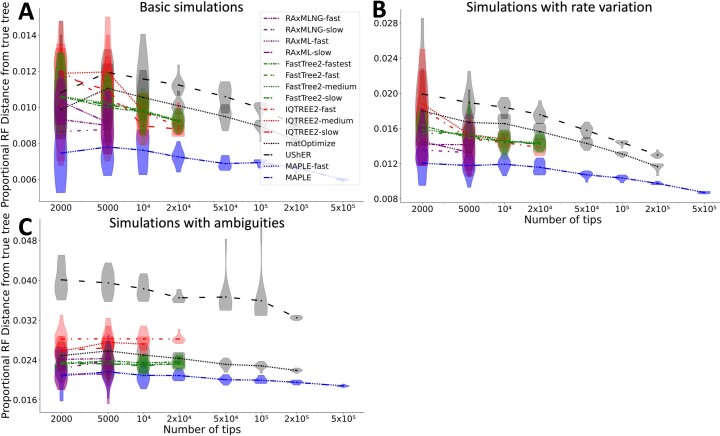
Comparison of proportional Robinson-Foulds distances of inferred trees from the correct simulated trees. On the Y axis we show the proportional Robinson-Foulds distances (that is, normalized by 2(*m* − 3) with *m* the number of samples in the tree) of the tree estimated by each method with respect with the true simulated tree of the corresponding scenario and replicate. We collapsed tree branches of the simulated trees where no mutation event was simulated. Trees were compared as unrooted, and polytomies were compared as such (we collapsed branches of inferred trees with length equal to the minimum allowed length by the corresponding inference method). **A** Results for simulated datasets with no rate variation or ambiguity. **B** Results on simulated data with rate variation but no ambiguities. **C** Simulated data with no rate variation but with ambiguities. Other details are the same as in Extended Data Figure S1.

**Extended Data Fig. 4:**
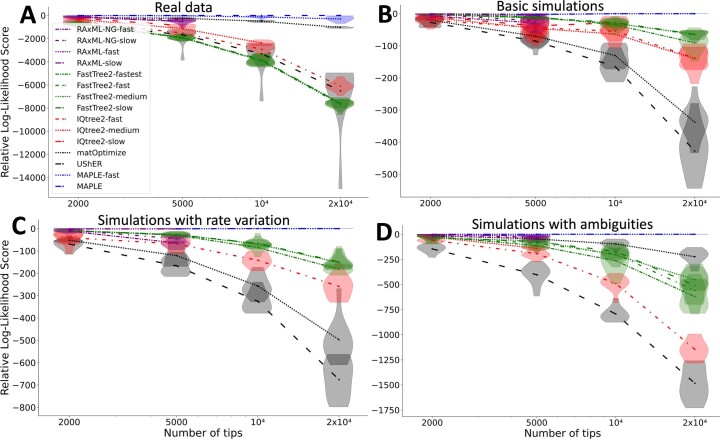
Comparison of relative likelihood scores of trees inferred by different phylogenetic methods from SARS-CoV-2 genomes. MAPLE leads to more accurate tree reconstruction (tree topologies with higher likelihoods) both in real data and simulations. On the Y axis we show the relative log-likelihood scores (computed with IQ-TREE 2) of the tree estimated by each method, as in Fig. 4f, with higher scores representing more likely tree estimates. **A** Results for subsamples from the real SARS-CoV-2 dataset. **B** Simulated datasets with no rate variation or ambiguity. **C** Results on simulated data with rate variation but no ambiguities. **D** Simulated data with sequence ambiguities but no rate variation. Other details are the same as in Extended Data Figure S1.

Maximum likelihood phylogenetic methods typically present trade-offs between accuracy and computational demand, with more accurate tree reconstruction requiring deeper, and therefore more time-consuming, tree space exploration. Thanks to the considerable time and memory savings brought by our approach to likelihood calculation, MAPLE can invest more resources in tree estimation than other methods, resulting in more accurate tree inference, while still requiring less time and memory than other maximum likelihood inference approaches (Fig. 4 and Extended Data Figs. 1–4).

**Fig. 5:**
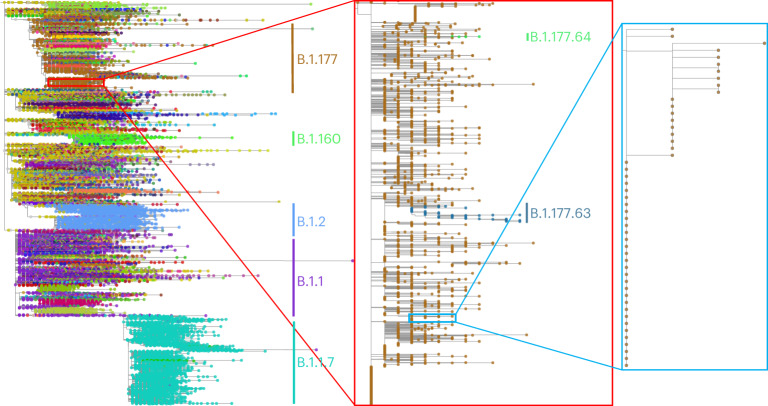
500,000-sample phylogeny inferred by MAPLE. Example phylogeny, with two consecutive zoom-ins each of about 100× magnification. Different SARS-CoV-2 lineages are shown in different colors, with some clades labeled to give context. Left—500,000-sample phylogeny estimated by MAPLE from real SARS-CoV-2 sequence data. Center—zoom-in on a subtree containing 3,600 B.1.177 samples. Right—further zoom-in on a subtree containing 49 samples. Phylogenies were plotted using Taxonium^45^.

As an example, MAPLE shows consistently higher accuracy than RAxML-NG^34^ (the most accurate of the methods we compared MAPLE against) on simulated and real SARS-CoV-2 datasets (Fig. 4c–f and Extended Data Figs. 3,4), while being more than 100-fold faster (Fig. 4a) and requiring less memory (Fig. 4b). MAPLE can also estimate trees about 25 times larger than IQ-TREE 2 (ref. ^24^) or FastTree 2 (ref. ^27^) (500,000 versus 20,000 samples) because of their 50-fold larger memory demand (Fig. 4b). Figure 5 shows an example of 500,000-sample SARS-CoV-2 whole-genome phylogeny, inferred by MAPLE v0.0.4 in 69.4 h with a maximum memory usage of 8.4 GB on one core of an Intel Xeon Gold 6252 Processor @ 2.10 GHz.

matOptimize^35^ (a recent feature improving the accuracy of UShER^28^) is a phylogenetic inference method that, similarly to MAPLE, has been tailored to the features of genomic epidemiological analyses, but that uses maximum parsimony rather than maximum likelihood principles. MAPLE shows similar computational demand to matOptimize, and less steep slopes in time and memory demand, therefore being able to estimate larger trees (Fig. 4a,b). matOptimize appears less accurate than maximum likelihood methods on simulated data (Fig. 4c–e) but more accurate on real data (Fig. 4f), being second only to MAPLE. A feature aiding the accuracy of matOptimize is its deep tree search, similar to MAPLE; an important disadvantage compared to maximum likelihood methods is instead its lack of a substitution model distinguishing different types of mutations (which we expect to have a bigger role with real data than in simulations due to the lower abundance of homoplasies in the latter). Combining both features helps MAPLE prevent hundreds of topological errors in simulated data (Fig. 4c–e) and, based on likelihood differences (Extended Data Fig. 4), we expect even more errors prevented with real data.

We can further improve the computational performance of MAPLE by reducing the depth of its tree space search; for example, using option ‘--fast’ in MAPLE, runtime typically becomes two to three times faster (Extended Data Fig. 1) without decreasing accuracy on simulated datasets (Extended Data Fig. 3) and while remaining the most accurate approach on real data (Extended Data Fig. 4).

**Fig. 6:**
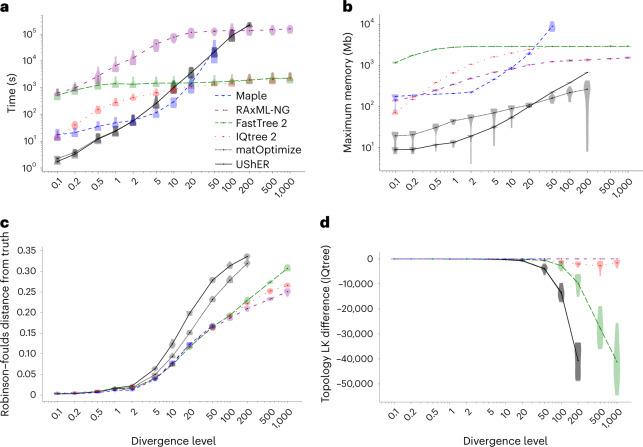
The relative performance of MAPLE deteriorates at higher levels of divergence. On data simulated with varying levels of divergence (*x*-axis values) relative to our baseline, the relative performance of methods tailored for pandemic-scale data (MAPLE and UShER/matOptimize) decreases with higher divergence. Here we always consider a dataset of 2,000 randomly sampled sequences (Methods). **a**, Time required to run each method. Each phylogenetic inference method considered is represented by a different color and line style (see legend). We ran each method up to the maximum divergence level that could be analyzed due to time (1 week) and memory limitations. Each violin plot summarizes values for ten replicates, and dots represent mean values. **b**, Maximum RAM demand required to run each method. **c**, Proportional Robinson–Foulds distances between estimated trees and true trees used in simulations. Higher values correspond to more errors in phylogenetic estimation. **d**, Log-likelihoods (LK) (computed with IQ-TREE 2) of phylogenies inferred by different methods, relative to the highest log-likelihood score obtained by any method for the same replicate. Higher values on the *y*-axis represent more likely estimates.

The approaches used in MAPLE are tailored for the scenario of many sequences at short divergence from each other. When considering datasets with higher divergence, we find that the performance of MAPLE deteriorates both in terms of time (Fig. 6a) and memory (Fig. 6b) demand; eventually, for datasets with about 50 times higher divergence than our baseline dataset (representing approximately 100 years of SARS-CoV-2 evolution), it becomes more feasible to use traditional maximum likelihood phylogenetic methods than MAPLE. For this reason, our software recommends the use of alternative methods at higher divergence levels (Methods). MAPLE’s accuracy remains however very high even at these levels of divergence—trees inferred from simulated data are similarly accurate as those of other maximum likelihood methods (Fig. 6c) and have the highest or near-highest likelihoods (Fig. 6d) for all the levels of divergence for which we could run MAPLE. While at higher divergence we expect MAPLE’s accuracy to deteriorate, it remains an accurate method for levels of divergence for which it can be used feasibly.

### Discussion

By developing an approximate alternative to the classic Felsenstein pruning algorithm, by including features of parsimony-based phylogenetic inference in a likelihood-based context and by using more concise data representation, we have achieved substantial reductions in memory and time demand and increases in accuracy compared to popular maximum likelihood approaches when inferring SARS-CoV-2 phylogenies. This enables state-of-the-art phylogenetic inference to be performed on larger datasets than previously possible.

Beyond SARS-CoV-2, our approach will be equally useful in any analysis with many sequences and with short evolutionary distances, such as in most scenarios in genomic epidemiology. This includes genomic datasets with many samples from an individual pathogen, including, for example, large collections of *Mycobacterium tuberculosis* genomes^36^ or influenza genomes^37^, and collections of genomic data from possible future pandemics. Our approach could also be combined with divide-and-conquer phylogenetic algorithms^38,39^ to further improve its performance and applicability. Other improvements, such as implementations in more efficient programming languages, sorting of genome positions (for example, ref. ^40^), and representation of ancestral genomes in terms of differences with respect to genomes at neighboring phylogenetic nodes (ref. ^35^) could further extend MAPLE’s applicability.

While in this work we have discarded inserted genetic material in genome sequences, it is possible, although not optimally efficient or informative, to consider insertions in a MAPLE phylogenetic analysis by including them as part of the reference sequence. In the future, it could be possible to efficiently represent insertions in MAPLE format and extend the algorithm and model to account for indel events.

The applicability of our methods goes beyond maximum likelihood phylogenetics. The same algorithms and data structures in MAPLE could also be used in a Bayesian setting because Bayesian phylogenetic methods (for example, BEAST^41,42^) use the same genetic data (multiple sequence alignments) and the same likelihood calculation algorithms as maximum likelihood phylogenetic methods, and so would benefit from the same reduction in computational demands. MAPLE’s speed could also enable the use of other techniques to assess phylogenetic uncertainty (such as the transfer bootstrap^43^ and approximate likelihood ratio tests^44^) on large datasets.

For these reasons, we expect that in the future, MAPLE and its algorithms will expand the computational toolkit of genomic epidemiology and could improve our preparedness for combating future epidemics.

## Methods

### Representation of genomic epidemiological sequence data

We use a concise and human-readable format for representing an alignment of closely related genome sequences, which we call MAPLE format. We express each genome sequence in terms of its differences (substitutions and deletions) with respect to the reference. We also record ambiguous positions (IUPAC ambiguity characters) and deleted or nonsequenced portions of the genomes (gap ‘-’ and ‘N’ characters, respectively).

As an illustrative example, we consider a reference genome ‘Reference’ comprising 20 ‘A’ characters:

>Reference

AAAAAAAAAAAAAAAAAAAA

(here represented in Fasta format). If a sampled genome ‘Sample’ consists of the sequence:

>Sample

NNNNNAAAAA---AAAAATA

when aligned to the reference, as it would be represented in Fasta format, we instead represent it as:

>Sample

N 1  5

-  11  3

T  19

where, in each entry (row), the first column represents the type of difference with respect to the reference, the second column in each row represents the position (along the reference genome) of the difference and the third column (which we only require for ‘N’ and ‘-’ entries) represents how many consecutive positions have this same character.

### Representation of ancestral sequences and likelihoods

In addition to representing sequence data at lower memory cost, we also calculate and represent partial likelihoods—probabilities of nucleotides at internal nodes of the tree—at low memory and time cost. For a genome of length *L* and a tree *ϕ* with ∣*ϕ*∣ nodes, we typically would need to compute and store 4 × *L* × ∣*ϕ*∣ such likelihoods. Instead, we replace partial likelihood vectors with more concise structures that we call ‘genome lists’.

Each entry of a genome list represents relative (normalized) phylogenetic partial likelihoods for either one position of the genome or for a set of consecutive positions that share similar features. An entry of type ‘A’, ‘C’, ‘G’ or ‘T’ represents an individual genome position where the nucleotide indicated, different from the reference genome nucleotide at the position, has a much higher likelihood than the other nucleotides. An entry of type ‘R’ represents a collection of contiguous sites with likelihood concentrated at the reference nucleotide. An entry of type ‘N’ indicates contiguous sites that contain no descendant sequence information. Finally, an entry of type ‘O’ (‘other’) indicates a position where multiple nucleotides have non-negligible relative partial likelihoods—in this case, all four likelihoods are stored as part of the entry.

Each entry also has a position element, identifying the genome position(s) it refers to, and a branch length element specifying the phylogenetic distance from the node the entry refers to. See Supplementary Methods Section S1.1 for a more in-depth description and examples.

### Calculation of genome lists

We described above and in Supplementary Methods S1.1 how we initialize genome lists for terminal nodes (samples) of the tree. Similar to the Felsenstein pruning algorithm, we calculate the genome list of an internal node only after calculating it for its children.

As is standard in phylogenetics, we assume that sequence evolution is a continuous-time and finite-space homogeneous Markov process, where all sites evolve independently^46^. We assume a nucleotide substitution process determined by a substitution rate matrix *Q* whose entries *q*_*XY*_, for any *X* ≠ *Y*, represent instantaneous rates of substitution of nucleotide *X* to nucleotide *Y*, and *q*_*XX*_ = − ∑_*Y*≠*X*_*q*_*XY*_. Transition probabilities over a branch length *l* are typically calculated using matrix exponentiation^46^; instead, considering the short branch lengths involved in genomic epidemiology, we use a first-order approximation:

1 $$P(Y| X,l)={e}^{lQ}\approx I+lQ$$

where *I* is the identity matrix. This means that the probability *P*(*Y*∣*X*,*l*) of nucleotide *X* evolving into nucleotide *Y* ≠ *X* is approximated as *lq*_*XY*_, and that *P*(*X*∣*X*,*l*) ≈ 1 + *lq*_*XX*_. Note that these will only be good approximations as long as the considered branch lengths are short, while for larger branches, these approximations will not be reliable. MAPLE warns the user if any estimated branch length is >0.01 (and if any genome has divergence >10% from the reference, due to the likely effect on computational demand), recommending instead the use of other methods.

**Extended Data Fig. 5:**
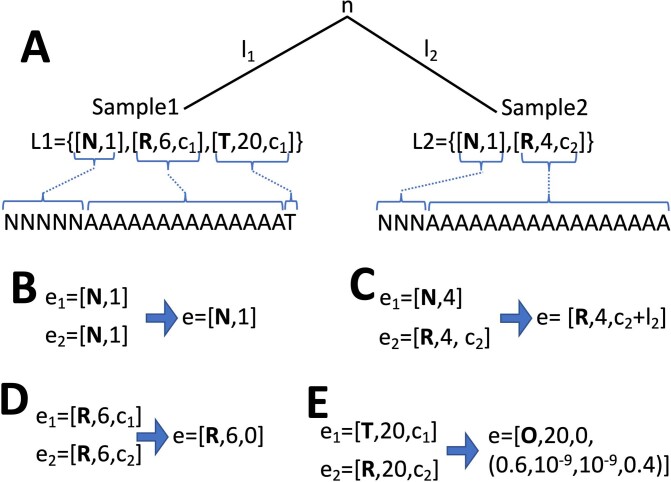
Graphical example of the merging of genome lists. We consider, as in the main text, an example reference genome of 20 “A” nucleotides, partial likelihood genome lists *L1* and *L2*, and a two-tips phylogeny with a branch long *l1* leading to Sample1 and *L1*, and one long *l2* leading to Sample2 and *L2*. **A** Graphical representation of the phylogeny, genomes, and partial likelihood genome lists. Blue parentheses and lines highlight the correspondence between genome list entries and portions of the observed genomes. We want to show how we calculate the partial likelihood genome list for the most recent common ancestor *n* of Sample1 and Sample2. Parameters *c1* and *c2* are always 0 for tree tips, but we keep the notation more general since for internal nodes these values can be strictly positive. **B** For the first three positions, both genomes contain no information (the corresponding genome list entries are of type **N**), so the parent node genome list entry is of type **N**. **C** For positions 4 and 5, Sample1 provides no information while Sample2 presents the reference allele. The corresponding parent node genome list entry is then of type **R** and its branch length element is *c2* + *l2*, the evolutionary distance between *n* and the last visited node in the tree with no state uncertainty at the considered positions. **D** From positions 6 to 19 both child node genome list entries are of type **R**. The corresponding genome list entry for *n* is then also of type **R**, and its branch length element is 0, which is the same as considering the reference alleles observed exactly at the parent node. **E** At the last position of the genome we observe “T” at Sample1 and reference nucleotide “A” at Sample2. The corresponding parent node genome list entry is then of type **O**. This entry also contains an explicit partial likelihood vector with the relative likelihoods of all four nucleotides. The branch length element of the entry is 0, since the relative partial likelihoods refer to *n*.

If different genome positions all belong to the same genome list entries in the two child nodes of node *n*, then they can all be represented by the same type of genome list entry for *n*. We exploit this fact by first finding such contiguous segments of genome positions, and then calculating genome list entries for *n*, one for each such segment. We can calculate each genome list entry in constant time, and so, at the short levels of divergence considered here, genome lists can be calculated much faster than classical phylogenetic likelihoods, which typically require linear time in genome size. The algorithm we use to calculate genome lists is described in detail in Supplementary Methods S1.2, and graphical examples are given in Extended Data Fig. 5.

### Other partial likelihoods

Partial likelihoods representing the probabilities of nucleotides conditional on all their observed descendants are normally sufficient for phylogenetic inference. However, when using a nonstationary model, additional types of likelihoods are useful^47^. Here we also use these additional likelihoods and represent them with additional genome lists. Furthermore, for most nodes of the tree, we also calculate genome lists representing relative likelihoods considering all the data in the alignment, which correspond to ancestral state reconstructions^48^. We present the details of these genome lists in Supplementary Methods S1.3.

### Phylogenetic inference

We infer phylogenies in two steps. First, we infer a starting tree by stepwise addition^33^—we start from a tree containing only one sample and iteratively expand it by adding (‘placing’) samples on it one at a time (Supplementary Methods S1.4). Then, we improve the starting tree topology using custom subtree pruning and regrafting^33^ (‘SPR’) proposals (Supplementary Methods S1.10).

Both initial sample placements and SPR searches are made in such a way as to focus on nodes of the tree that are most promising for beneficial placements and SPR proposals (Fig. 3). The likelihood benefit of placements and SPR moves can be calculated quickly using our precomputed genome lists (Supplementary Methods S1.5). Also, every time we modify the tree, we only need to update the genome lists of a small portion of the tree (Supplementary Methods S1.7).

During estimation of the initial tree, we also estimate the substitution model (Supplementary Methods S1.9).

### Software implementation

We implemented our methods in a Python3 script available from https://github.com/NicolaDM/MAPLE. For efficiency, we recommend its execution with the pypy3 implementation of Python (https://www.pypy.org/#!).

### Other phylogenetic methods considered

We compare the performance of MAPLE to high-performance and popular maximum likelihood phylogenetic methods that are often used to analyze large sequence datasets as follows: IQ-TREE v2.1.3 (ref. ^24^), FastTree v2.1.11 (ref. ^27^) (double precision, no SSE3) and RAxML-NG v1.0.2 (ref. ^34^). For all these methods, we adopt a GTR substitution model^49^. We also consider the parsimony-based method matOptimize v0.5.1 (ref. ^35^), a recent approach to improving the accuracy of UShER^28^ trees, which has been tailored for SARS-CoV-2 datasets. We selected program options to permit a fair comparison of methods, with each being tuned to the largest problems it could analyze on available hardware. In detail:

We ran IQ-TREE 2 with options ‘-quiet’ to reduce screen output, ‘-nt 1’ to use only one core per replicate on our cluster and ‘-fast’, with which only nearest neighbor interchange (NNI) moves are used. For simulations with rate variation, we used a GTR+G model.

FastTree 2 was executed with options ‘-quiet’ to limit screen output, ‘-nosupport’ to skip support value computations and ‘-nocat’ to ignore rate variation (except for simulations with rate variation, for which we use ‘-cat 4’). We also used option ‘-fastest’ to reduce the time demand of NNI steps.

RAxML-NG was run with options ‘--threads 1’ to use only one core per replicate on our cluster, ‘--blmin 0.000005’ to increase the minimum branch length considered and ‘--tree pars{1}’ to start the tree search from a parsimony tree. For simulations with rate variation, we used a GTR+G model.

UShER v0.5.1 and matOptimize were run with option ‘-T 1’ to use a single thread per replicate and were run using the vcf input file format (option ‘-v’). matOptimize was run starting from the initial tree estimate of UShER and using option ‘-n’ to avoid the creation of intermediate files.

We ran MAPLE with default parameters and using PyPy (v7.3.5 with GCC 7.3.1 20180303 for Python 3.7.10; see https://www.pypy.org/#!).

Additional options considered for these and additional methods are described in Supplementary Methods Section S1.11, with corresponding results reported in Extended Data Figs. 1–4.

### Real SARS-CoV-2 sequence data

We randomly subsampled, without replacement, a given number of sequences from the 540,520 whole genomes that were represented both in the 31 March 2021 global unmasked SARS-CoV-2 alignment from GISAID^37^ and in the corresponding phylogenetic tree (https://www.gisaid.org/). No ethical approval was required to access or analyze this data. We did not mask sites or filter out sequences. We use the consensus of all the sequences in the global GISAID alignment as reference genome for MAPLE. When measuring running times, we did not consider the cost of creating the input alignment for a given method.

### Simulated SARS-CoV-2 sequence data

For real datasets, we have the drawback of not knowing the true underlying phylogenetic tree, which makes it harder to assess the accuracy of different phylogenetic inference methods. For this reason, we also simulated SARS-CoV-2 alignments of known phylogeny and substitution dynamics. We used the publicly available 26 October 2021 global SARS-CoV-2 phylogenetic tree as background ‘true’ tree from http://hgdownload.soe.ucsc.edu/goldenPath/wuhCor1/UShER_SARS-CoV-2/ (ref. ^21^), representing the evolutionary relationship of 2,250,054 SARS-CoV-2 genomes as obtained using UShER^28^. We used phastSim v0.0.3 (ref. ^50^) to simulate sequence evolution along this tree according to SARS-CoV-2 nonstationary neutral mutation rates^51^ and using the SARS-CoV-2 Wuhan-Hu-1 genome^52^ as root sequence. We simulated three different scenarios:

- The ‘basic’ simulation scenario (no rate variation and full genomes available).
- The ‘rate variation’ scenario, where we allow different genome positions to evolve at different speeds in our simulations to mimic the effect on genome evolution of variable mutation rates and selective pressures along the genome. We simulated four genome site categories, all with the same frequency and with relative substitution rates of 0.1, 0.5, 1 and 2.
- The ‘sequence ambiguity’ scenario, where we modified the simulated sequence data of the basic simulation scenario to include ambiguous characters. To realistically mimic amplicon drop-out effects^53^, for each simulated sequence, we sample one random sequence from the real dataset and copy-paste from it the stretches of ‘N’ and gap ‘-’ characters into the simulated sequence. Additionally, because contamination and mixed infections can result in individual ambiguous characters specifically at phylogenetically informative sites of the genome^54^, we count the number of isolated ambiguous characters in the real sequence, and we mask an equal number of randomly selected SNPs (differences with respect to the reference genome) in the simulated sequence. If more isolated ambiguous characters are observed in the real sequence than SNPs in the simulated sequence, then we simply mask all SNPs in the simulated sequence.

We also created a second set of simulations to assess the effect of different levels of divergence on MAPLE’s phylogenetic inference. First, we took a random 10,000-sample subtree of the phylogeny above. We then simulated genome evolution along this tree as in the ‘basic’ scenario above, but scaling the branch lengths of the tree by different divergence factors ranging from 0.1 to 1000. For each such simulated alignment, we then sampled 2,000 random sequences for each of 10 replicates for each divergence scaling factor; in these simulations, we used MAPLE v0.2.0, while for the other analyses, we used v0.0.4.

### Comparison of methods’ performance

We measured the computational demand of different approaches in estimating phylogenies by tracking the running time and maximum memory demand of all methods. All methods were run in parallel, assigning one thread per replicate per method. Because matOptimize requires an initial run of UShER, the running time of matOptimize is defined as the sum of the time it took to execute UShER followed by matOptimize; the maximum memory demand for matOptimize was defined as the highest of the maximum memory demands of the two methods.

We used two methods to compare the topological inference accuracy of different approaches. The first compares the likelihoods of the estimated tree topologies. Trees with higher topology likelihoods are interpreted as better estimates. Because the phylogenetic likelihood of the same tree computed by different software can differ due to different approximations employed, we use the same software, IQ-TREE 2, to calculate the likelihood of the topologies inferred by all methods. To make the comparison of topological accuracy of different methods even fairer, in particular considering that maximum parsimony methods UShER and matOptimize do not represent branch lengths in the same way as maximum likelihood methods and do not estimate substitution models, when measuring topology tree likelihoods we run IQ-TREE 2 using the tree to be assessed as starting tree, and performing model and branch length optimization but without attempting topological improvements. In simulations with rate variation, we run IQ-TREE 2 with a GTR+G model with four categories; otherwise, we use a plain GTR model. Note that the use of IQ-TREE 2 for tree topology likelihood estimation limits the size of the trees that can be assessed due to the memory demand of the software.

The second measurement of phylogenetic accuracy (only available for simulated data for which the correct tree is known) is to calculate the Robinson–Foulds distance^55^ between an inferred tree and the corresponding true simulated tree. This distance gives a measure of how topologically close an inferred tree is to the true tree, and therefore quantifies inference error. We consider trees as unrooted, collapse all branches of the simulated trees on which no simulated mutation events occurred, and collapse all branches shorter than a minimum branch length (defined by the minimum branch length considered by each estimation method) so as to represent trees as multifurcating when a method finds little or no support for the local branching order. Robinson–Foulds distance calculations were performed with a custom implementation of Day’s algorithm^56^.

### Statistics and reproducibility

The size and composition of the datasets considered were determined by the availability of SARS-CoV-2 genome alignments and phylogenetic trees and the capabilities of different methods to analyze these data; no statistical method was used to predetermine sample size. Subsample sizes (ranging from 2,000 to 500,000) were chosen to showcase the performance of the methods considered at different dataset sizes. All subsamples were generated uniformly at random, and the analysis can be replicated using our scripts in https://github.com/NicolaDM/MAPLE.

### Reporting summary

Further information on research design is available in the Nature Portfolio Reporting Summary linked to this article.

## Online content

Any methods, additional references, Nature Portfolio reporting summaries, source data, extended data, supplementary information, acknowledgements, peer review information; details of author contributions and competing interests; and statements of data and code availability are available at 10.1038/s41588-023-01368-0.

## Supplementary information

Supplementary Information Supplementary Methods.

Reporting Summary

## Data Availability

All real data was downloaded from the GISAID initiative website (https://www.gisaid.org/, 31 March 2021 alignment, accessed from https://www.epicov.org/epi3/) which requires a GISAID account and acceptance of the GISAID data sharing conditions. Unique identifiers of the samples used are listed in the file https://github.com/NicolaDM/MAPLE/blob/main/2021-03-31_unmasked_differences_reduced_namesOnly.txt.zip.

## Code availability

The code is available from https://github.com/NicolaDM/MAPLE ref. ^57^.
