# Supplementary material for: Maximum likelihood pandemic-scale phylogenetics
Source: Nat Genet. 2023 Apr 10;55(5):746–52. doi: 10.1038/s41588-023-01368-0 (PMC10181937; doi:10.1038/s41588-023-01368-0)
Supplement: Supplementary file 2 — Reporting Summary [file 41588_2023_1368_MOESM2_ESM.pdf]

nature portfolio

Corresponding author(s): Nicola De Maio
Last updated by author(s): Jan 26, 2023

Reporting Summary

Nature Portfolio wishes to improve the reproducibility of the work that we publish. This form provides structure for consistency and transparency in reporting. For further information on Nature Portfolio policies, see our Editorial Policies and the Editorial Policy Checklist.

Statistics

For all statistical analyses, confirm that the following items are present in the figure legend, table legend, main text, or Methods section.

n/a	Confirmed	
☐	☒	The exact sample size (n) for each experimental group/condition, given as a discrete number and unit of measurement
☒	☐	A statement on whether measurements were taken from distinct samples or whether the same sample was measured repeatedly
☒	☐	The statistical test(s) used AND whether they are one- or two-sided<br>*Only common tests should be described solely by name; describe more complex techniques in the Methods section.*
☒	☐	A description of all covariates tested
☒	☐	A description of any assumptions or corrections, such as tests of normality and adjustment for multiple comparisons
☐	☒	A full description of the statistical parameters including central tendency (e.g. means) or other basic estimates (e.g. regression coefficient) AND variation (e.g. standard deviation) or associated estimates of uncertainty (e.g. confidence intervals)
☒	☐	For null hypothesis testing, the test statistic (e.g. F, t, r) with confidence intervals, effect sizes, degrees of freedom and P value noted<br>*Give P values as exact values whenever suitable.*
☒	☐	For Bayesian analysis, information on the choice of priors and Markov chain Monte Carlo settings
☒	☐	For hierarchical and complex designs, identification of the appropriate level for tests and full reporting of outcomes
☒	☐	Estimates of effect sizes (e.g. Cohen's d, Pearson's r), indicating how they were calculated

Our web collection on statistics for biologists contains articles on many of the points above.

Software and code

Policy information about availability of computer code

Data collection	No software was used for data collection.
Data analysis	The software described in our manuscript is deposited on GitHub: https://github.com/NicolaDM/MAPLE .<br>Other software used in the manuscript is also open source:<br>phastSim v0.0.3 https://github.com/NicolaDM/phastSim ;<br>IQ-TREE v2.1.3 https://github.com/iqtree/iqtree2 ;<br>FastTree v2.1.11 http://www.microbesonline.org/fasttree/ ;<br>RAxML-NG v1.0.2 https://github.com/amkozlov/raxml-ng ;<br>RAxML v8.2.11 https://github.com/stamatak/standard-RAxML ;<br>UShER and matOptimize v0.5.1 https://github.com/yatisht/usher

For manuscripts utilizing custom algorithms or software that are central to the research but not yet described in published literature, software must be made available to editors and reviewers. We strongly encourage code deposition in a community repository (e.g. GitHub). See the Nature Portfolio guidelines for submitting code & software for further information.

Data

Policy information about availability of data

All manuscripts must include a data availability statement. This statement should provide the following information, where applicable:

- Accession codes, unique identifiers, or web links for publicly available datasets
- A description of any restrictions on data availability
- For clinical datasets or third party data, please ensure that the statement adheres to our policy

All real data used in this manuscript was downloaded from the GISAID initiative website (https://www.gisaid.org/ , 31 March 2021 alignment, accessed from https://www.epicov.org/epi3/) which requires a GISAID account and acceptance of the GISAID data sharing conditions. Unique identifiers of the samples used in the manuscript are listed in the file https://github.com/NicolaDM/MAPLE/blob/main/2021-03-31_unmasked_differences_reduced_namesOnly.txt.zip .

Human research participants

Policy information about studies involving human research participants and Sex and Gender in Research.

Reporting on sex and gender	Not applicable.
Population characteristics	Not applicable.
Recruitment	Not applicable.
Ethics oversight	Not applicable.

Note that full information on the approval of the study protocol must also be provided in the manuscript.

Field-specific reporting

Please select the one below that is the best fit for your research. If you are not sure, read the appropriate sections before making your selection.

☐ Life sciences ☐ Behavioural & social sciences ☒ Ecological, evolutionary & environmental sciences

For a reference copy of the document with all sections, see nature.com/documents/nr-reporting-summary-flat.pdf

Ecological, evolutionary & environmental sciences study design

All studies must disclose on these points even when the disclosure is negative.

Study description	Development of new large-scale phylogenetic methods. Only in silico analyses were performed.
Research sample	SARS-CoV-2 genome data was downloaded from GISAID.
Sampling strategy	All available data was downloaded, and subsamples were created by sampling genomes uniformly at random.
Data collection	The 31 March 2021 Genome alignment was downloaded from GISAID.
Timing and spatial scale	Not applicable.
Data exclusions	We only considered genomes present in the GISAID phylogeny to avoid partial genomes.
Reproducibility	All the software we used is open source, the data is available on GISAID, and we describe all the options we used for each software in the manuscript.
Randomization	Not applicable.
Blinding	Not applicable.

Did the study involve field work? ☐ Yes ☒ No

Reporting for specific materials, systems and methods

We require information from authors about some types of materials, experimental systems and methods used in many studies. Here, indicate whether each material, system or method listed is relevant to your study. If you are not sure if a list item applies to your research, read the appropriate section before selecting a response.

Materials & experimental systems

n/a	Involved in the study
☒	☐ Antibodies
☒	☐ Eukaryotic cell lines
☒	☐ Palaeontology and archaeology
☒	☐ Animals and other organisms
☒	☐ Clinical data
☒	☐ Dual use research of concern

Methods

n/a	Involved in the study
☒	☐ ChIP-seq
☒	☐ Flow cytometry
☒	☐ MRI-based neuroimaging
